# Rhinitis prevalence and incidence in a cohort of children with recurrent wheezing: from preschool age to adolescence

**DOI:** 10.1186/2045-7022-3-S2-P5

**Published:** 2013-07-16

**Authors:** Ana Pereira, Ângela Gaspar, Mário Morais-Almeida

**Affiliations:** 1 Hospital CUF-Descobertas, Immunoallergy Department, Lisbon, Portugal

## Background

The aims of this study were 1) to estimate baseline rhinitis prevalence in a cohort of preschool children with recurrent wheezing (RW), 2) to assess personal and socio-demographic factors associated with rhinitis in wheezers, and 3) to estimate rhinitis incidence over a 13 years follow-up period.

## Methods

This cohort study included 308 children observed as first appointments in a tertiary hospital's outpatient clinic in 1993. All children aged <7 years old and with RW (≥3 wheezing episodes responsive to bronchodilator in the previous year with symptom-free intervals in-between) were included. Evaluations included an allergy consultation and skin-prick tests (SPT). In 1993, the participant's mean(SD) age was 3.7 (1.7) years, 61% were male, 48% had positive SPT, 22% had personal history of atopic eczema and 6% of food allergy. Children were reassessed in 2001 (n=249) and 2006 (n=170); no significant differences were found between the characteristics of the children assessed in each evaluation (p>0.49). A multiple logistic regression model was developed to study risk factors for rhinitis at preschool age; results were presented as odds ratio (OR) with 95% confidence interval (CI).

## Results

In 1993, the prevalence of rhinitis was 60% (95%CI[55-65]). Rhinitis at preschool age was positively associated with atopy (19.6[9.1-42.4]), maternal asthma (2.6[1.1-6.3]), personal history of food allergy (13.2[(1.2-120.6)]) and atopic eczema (2.6[1.1-6.4]); kindergarten attendance before the age of 12 months presented a negative association (0.4[0.2-0.8]). Recurrent respiratory infections, parental rhinitis and paternal asthma were not significantly associated with rhinitis diagnosis. When considering the period from 1993 to 2001, 10 children (out of the 93 previously without rhinitis) had new rhinitis diagnosis (incidence: 11%). The 1993-2001 incidence was highest in atopic children (33% vs. 7% in non-atopic, p=0.007). Most of the children with rhinitis at preschool age persisted with rhinitis diagnosis in 2001 (96%). In 2001-2006, 11 new rhinitis cases (out of 65) were reported (incidence: 17%); the incidence was also highest in atopic children (56%). In 2006, 87% of those with rhinitis had it since preschool age.

## Conclusion

The prevalence of rhinitis in preschool children with recurrent wheezing was high and atopy presented the strongest association with rhinitis diagnosis. Most of the children with rhinitis in adolescence had rhinitis since preschool age.

